# Supplementary material for: Finding genetically-supported drug targets for Parkinson’s disease using Mendelian randomization of the druggable genome
Source: Nat Commun. 2021 Dec 20;12:7342. doi: 10.1038/s41467-021-26280-1 (PMC8688480; doi:10.1038/s41467-021-26280-1)
Supplement: Supplementary file 1 — Supplementary Information [file 41467_2021_26280_MOESM1_ESM.pdf]

Supplementary Information

Contents

Supplementary figures **2**

Supplementary data dictionary **3**

Supplementary figures

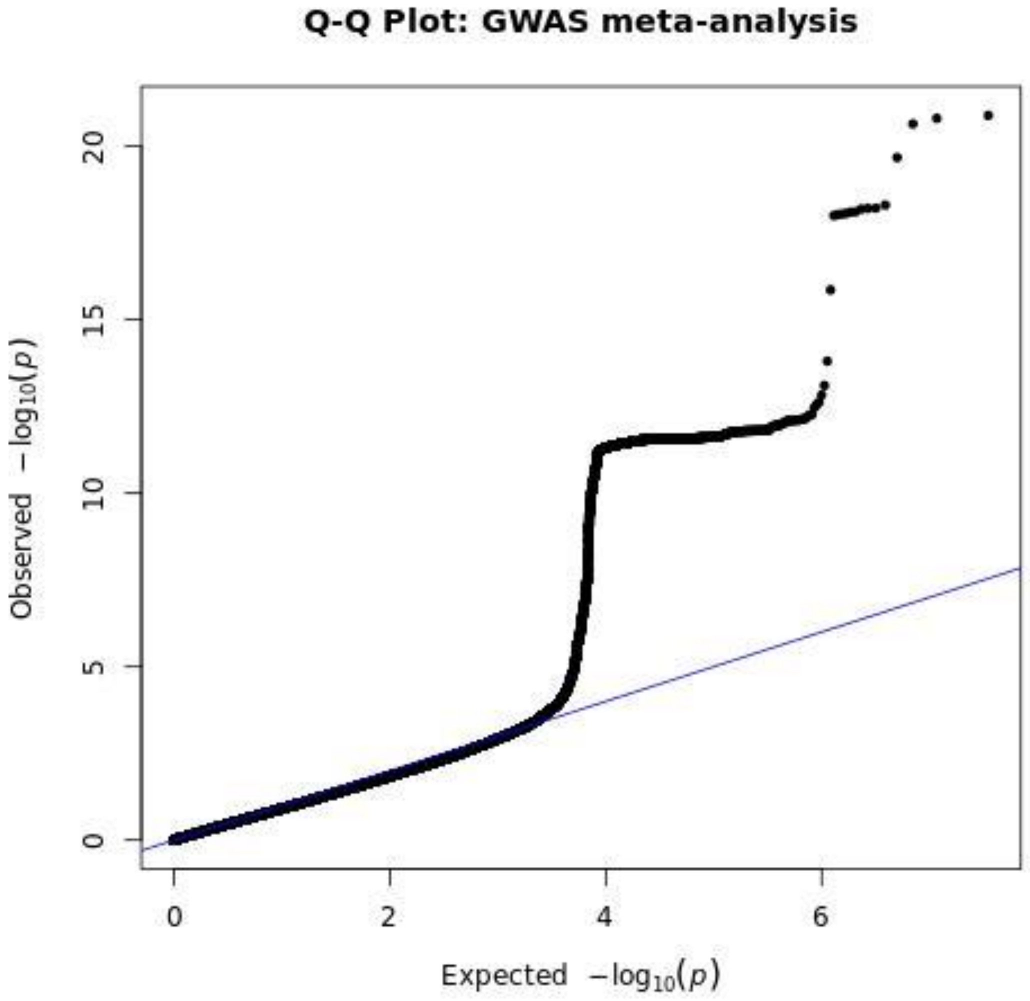

Supplementary Fig. 1 | Quantile-quantile plot Parkinson's disease replication cohort. Scatter plot of the observed versus expected -$\log_{10}$(p-values) generated by the meta-analysis of case-control genome-wide association study data used for the replication step for Parkinson's disease risk.

Supplementary data dictionary

Below are definitions and descriptions for column headings used in supplementary tables.

beta: MR effect size.

beta_lci95: Lower boundary of 95% confidence interval of the MR effect size (beta).

beta_uci95: Upper boundary of 95% confidence interval of the MR effect size (beta).

chr_name: Chromosome of the gene tested.

clump_tresh: Clumping threshold used

cochrans_q: Cochran's Q.

cochrans_q_pval: Cochran's Q p-value.

druggability_tier: Druggability tier for the gene as defined by Finan et al. 2017 (PMID: 28356508).

1 = genes encoding targets of approved and clinical-phase drugs. 2 = genes encoding proteins that are targeted by small, drug-like molecules in vivo, and genes that are highly similar to approved drug targets. 3 and 4 = genes encoding extracellular or secreted proteins, genes with some similarity to approved drug targets, and genes which belong to key druggable gene families, if not already included in tiers 1 and 2. Of these, genes in tier 3 were in within ±50 kilobases of a GWAS SNP and encoded an extracellular protein. The remainder were put in tier 4."

egger_intercept: MR-Egger intercept.

egger_intercept_95_ci: 95% confidence intervals of the MR-Egger intercept.

egger_intercept_pvalue: P-value of the MR-Egger intercept.

exposure: Name of the gene tested.

fdr_qval: False discovery rate-corrected p-value (also called a q-value).

gene_end: Base pair position of gene end.

gene_start: Base pair position of gene start.

i2: I^2 value

method: Method used to calculate the MR effect estimate.

nsnp: Number of SNPs used for the MR calculation.

or: Odds ratio.

or_lci95: Lower boundary of 95% confidence interval of the MR effect size (odds ratio).

or_uci95: Upper boundary of 95% confidence interval of the MR effect size (odds ratio).

outcome: Name of the outcome tested.

p: Raw p-value.

p1: Colocalization data; prior probability that a SNP in the tested region is significantly associated with expression of the tested gene.

p12: Colocalization data; prior probability that a SNP in the tested region is significantly associated with both expression of the tested gene and the tested PD outcome.

p2: Colocalization data; prior probability that a SNP in the tested region is significantly associated with expression of the tested PD outcome.

PP.H0.abf: Colocalization data; PPH0, posterior probability that there is no association with either trait.

PP.H1.abf: Colocalization data; PPH1, posterior probability that there is association with expression of the gene, but not the PD trait.

PP.H2.abf: Colocalization data; PPH2, posterior probability that there is association with the PD trait, but not expression of the gene.

PP.H3.abf: Colocalization data; PPH3, posterior probability that there is association with the PD trait and expression of the gene, with distinct causal variants.

PP.H4.abf: Colocalization data; PPH4, posterior probability that there is association with the PD trait and expression of the gene, with a shared causal variant.

se: Standard error.

sum_pph3_pph4: Colocalization data; sum of PPH3 and PPH4. PPH3+PPH4 < 0.8 indicated low power for colocalization.

tissue_qtl_type: Tissue in which expression of the gene was measured (blood or brain), and the QTL type (expression of protein levels).
