# Supplementary material for: Finding genetically-supported drug targets for Parkinson’s disease using Mendelian randomization of the druggable genome
Source: Nat Commun. 2021 Dec 20;12:7342. doi: 10.1038/s41467-021-26280-1 (PMC8688480; doi:10.1038/s41467-021-26280-1)
Supplement: Supplementary file 3 — Description of Additional Supplementary Files [file 41467_2021_26280_MOESM3_ESM.pdf]

Description of Additional Supplementary Files

File Name: Supplementary Data 1
Description: Related to Fig. 2, Fig. 3 and Fig. 4. MR results for all genes reaching significance for PD risk (discovery phase and replication phase), age at onset and progression markers. The “method” column indicates the statistical test used to calculate each effect size, and the “fdr_qval” column indicates p value corrected for the number of genes tested.

File Name: Supplementary Data 2
Description: Related to Fig. 2, Fig. 3 and Fig. 4. MR quality control (MR Egger intercept, Cochran’s Q, $/^2$ tests) results for all genes reaching significance for PD risk (discovery phase and replication phase), age at onset and progression markers.

File Name: Supplementary Data 3
Description: Related to Fig. 2, Fig. 3 and Fig. 4. MR results for all genes tested for PD risk (discovery phase and replication phase), age at onset and progression markers. The “method” column indicates the statistical test used to calculate each effect size, and the “fdr_qval” column indicates p value corrected for the number of genes tested.

File Name: Supplementary Data 4
Description: Related to Fig. 2, Fig. 3 and Fig. 4. MR quality control results (MR Egger intercept, Cochran’s Q, $/^2$ tests) for all genes tested for PD risk (discovery phase and replication phase), age at onset and progression markers.

File Name: Supplementary Data 5
Description: Related to Fig. 5. MR results for all proteins where a pQTL was available. The “method” column indicates the statistical test used to calculate each effect size, and the “fdr_qval” column indicates p value corrected for the number of genes tested.

File Name: Supplementary Data 6
Description: Related to Fig. 5. MR quality control results (MR Egger intercept, Cochran’s Q, $/^2$ tests) for all proteins where a pQTL was available.

File Name: Supplementary Data 7
Description: Related to Fig. 2, Fig. 3 and Fig. 4. Colocalization results for all exposures with sufficient power ($PPH3 + PPH4 \geq 0.8$).

File Name: Supplementary Data 8
Description: MR results for genes that reached significance in the main PD risk, age at onset and progression analyses, removing SNPs associated with the expression of more than one gene.

File Name: Supplementary Data 9
Description: MR quality control results (MR Egger intercept, Cochran’s Q, $/^2$ tests) for genes that reached significance in the main PD risk, age at onset and progression analyses, removing SNPs associated with the expression of more than one gene.
