# Supplementary material for: Finding genetically-supported drug targets for Parkinson’s disease using Mendelian randomization of the druggable genome
Source: Nat Commun. 2021 Dec 20;12:7342. doi: 10.1038/s41467-021-26280-1 (PMC8688480; doi:10.1038/s41467-021-26280-1)
Supplement: Supplementary file 13 — Reporting Summary [file 41467_2021_26280_MOESM13_ESM.pdf]

Corresponding author(s): Nicholas Wood

Last updated by author(s): Aug 5, 2021

Reporting Summary

Nature Research wishes to improve the reproducibility of the work that we publish. This form provides structure for consistency and transparency in reporting. For further information on Nature Research policies, see our Editorial Policies and the Editorial Policy Checklist.

Statistics

For all statistical analyses, confirm that the following items are present in the figure legend, table legend, main text, or Methods section.

n/a	Confirmed	
☐	☒	The exact sample size (n) for each experimental group/condition, given as a discrete number and unit of measurement
☐	☒	A statement on whether measurements were taken from distinct samples or whether the same sample was measured repeatedly
☐	☒	The statistical test(s) used AND whether they are one- or two-sided<br>*Only common tests should be described solely by name; describe more complex techniques in the Methods section.*
☒	☐	A description of all covariates tested
☐	☒	A description of any assumptions or corrections, such as tests of normality and adjustment for multiple comparisons
☐	☒	A full description of the statistical parameters including central tendency (e.g. means) or other basic estimates (e.g. regression coefficient) AND variation (e.g. standard deviation) or associated estimates of uncertainty (e.g. confidence intervals)
☐	☒	For null hypothesis testing, the test statistic (e.g. F, t, r) with confidence intervals, effect sizes, degrees of freedom and P value noted<br>*Give P values as exact values whenever suitable.*
☐	☒	For Bayesian analysis, information on the choice of priors and Markov chain Monte Carlo settings
☒	☐	For hierarchical and complex designs, identification of the appropriate level for tests and full reporting of outcomes
☐	☒	Estimates of effect sizes (e.g. Cohen's d, Pearson's r), indicating how they were calculated

Our web collection on statistics for biologists contains articles on many of the points above.

Software and code

Policy information about availability of computer code

Data collection	No software was used for data collection.
Data analysis	The code used for this study is openly available at: https://github.com/catherinestorm/mr_druggable_genome_pd. Data processing was completed using R software (version 3.6.3), with packages TwoSampleMR (version 0.5.4), MendelianRandomization (version 0.4.2), coloc (version 4.0.4), dplyr (version 1.0.0), readr (version 1.3.1), stringr (version 1.4.0), tidyverse (version 1.3.0), forestplot (version 1.9), plyr (version 1.8.6), devtools (version 2.3.0), and remotes (version 2.1.1). The GWAS meta-analysis was completed using METAL (version 2011-03-25). Effect estimates (beta, odds ratio) are provided in Figures 2, 3, 4, and 5, as well as in Supplementary Tables 1, 3, and 5. These were calculated as described in the methods section and indicated on the GitHub.

For manuscripts utilizing custom algorithms or software that are central to the research but not yet described in published literature, software must be made available to editors and reviewers. We strongly encourage code deposition in a community repository (e.g. GitHub). See the Nature Research guidelines for submitting code & software for further information.

Data

Policy information about availability of data

All manuscripts must include a data availability statement. This statement should provide the following information, where applicable:

- Accession codes, unique identifiers, or web links for publicly available datasets
- A list of figures that have associated raw data
- A description of any restrictions on data availability

The supplementary information contains the full results of this study. Tissue-specific eQTL data were obtained from the eQTLGen (https://eqtlgen.org/) and PsychENCODE consortia (http://resource.psychencode.org/). The druggable genome data were obtained from the original authors; an immediately accessible version of the druggable genome is available in the supplementary materials of the original publication (Finan et al 2017; PMID: 28356508). Subsets of the most

recent PD risk GWAS were obtained from the original authors (Nalls et al 2019; PMID: 31701892). GWAS summary statistics for PD age at onset are available on the IPDGC website (http://pdgenetics.org/resources). PD progression GWAS data can be found here: https://pdgenetics.shinyapps.io/pdprogmetagwasbrowser/.

Field-specific reporting

Please select the one below that is the best fit for your research. If you are not sure, read the appropriate sections before making your selection.

[x] Life sciences [ ] Behavioural & social sciences [ ] Ecological, evolutionary & environmental sciences

For a reference copy of the document with all sections, see nature.com/documents/nr-reporting-summary-flat.pdf

Life sciences study design

All studies must disclose on these points even when the disclosure is negative.

Sample size	To maximize the power of this Mendelian randomization study , we used the largest available GWAS data for each exposure and outcome.
Data exclusions	We used SNPs associated with the expression of 4,863 druggable genes (curated by co-author Chris Finan and Aroon Hingorani) in blood and brain tissue. We kept all SNPs with false discovery rate <0.05 and located within 5 kb of the associated gene. These were clumped at r2 = 0.2.
Replication	For PD risk, we replicated out analysis in an independent case-control cohort. We identified 23 pQTL studies, of which four studies (1) reported significant pQTLs in individuals of European descent for any of the druggable proteins proposed by our eQTL analysis, (2) provided all the SNP information required for MR and (3) reported SNPs that were available in our PD outcome data. If there was a protein quantitative trait locus available, we validated our findings using these pQTLs.
Randomization	This Mendelian randomization study is randomized at conception of each study participant, when genetic alleles are independently allocated.
Blinding	This study did not analyze any individual-level data, so blinding was not applicable. Study participants are unlikely to be aware of their own genotypes and the effect of a genotype on the exposures, meaning that individuals of the are effectively blinded.

Reporting for specific materials, systems and methods

We require information from authors about some types of materials, experimental systems and methods used in many studies. Here, indicate whether each material, system or method listed is relevant to your study. If you are not sure if a list item applies to your research, read the appropriate section before selecting a response.

Materials & experimental systems

n/a	Involved in the study	
[x]	[ ]	Antibodies
[x]	[ ]	Eukaryotic cell lines
[x]	[ ]	Palaeontology and archaeology
[x]	[ ]	Animals and other organisms
[x]	[ ]	Human research participants
[x]	[ ]	Clinical data
[x]	[ ]	Dual use research of concern

Methods

n/a	Involved in the study	
[x]	[ ]	ChIP-seq
[x]	[ ]	Flow cytometry
[x]	[ ]	MRI-based neuroimaging
